# Effectiveness and mechanisms of combined use of antioxidant nutrients in protecting against oxidative stress‐induced neuronal loss and related neurological deficits

**DOI:** 10.1111/cns.14886

**Published:** 2024-07-28

**Authors:** Lu Wang, Yingjuan Wei, Zhenzhou Sun, Wenya Tai, Hui Li, Yaling Yin, Lin‐Hua Jiang, Jian‐Zhi Wang

**Affiliations:** ^1^ Henan Key Laboratory of Neurorestoratology The First Affiliated Hospital of Xinxiang Medical University Xinxiang China; ^2^ Department of Physiology and Pathophysiology, Sino‐UK Joint Laboratory of Brain Function and Injury of Henan Province, School of Basic Medical Sciences Xinxiang Medical University Xinxiang China; ^3^ The Third Affiliated Hospital of Xinxiang Medical University Xinxiang China; ^4^ Department of Blood Transfusion Xuchang Central Hospital Xuchang China; ^5^ School of Biomedical Sciences University of Leeds Leeds UK; ^6^ EA4245, Transplantation, Immunology and Inflammation, Faculty of Medicine University of Tours Tours France; ^7^ Key Laboratory of Ministry of Education of China for Neurological Disorders, Tongji Medical College Huazhong University of Science and Technology Wuhan China

**Keywords:** antioxidant nutrients, brain dysfunction, neurodegenerative disease, neuronal loss, oxidative stress

## Abstract

**Background:**

Oxidative stress is a well‐known pathological factor driving neuronal loss and age‐related neurodegenerative diseases. Melatonin, coenzyme Q10 and lecithin are three common nutrients with an antioxidative capacity. Here, we examined the effectiveness of them administrated individually and in combination in protecting against oxidative stress‐induced neuronal death in vitro, and neurodegenerative conditions such as Alzheimer's disease and associated deficits in vivo.

**Methods:**

Mouse neuroblastoma Neuro‐2a (N2a) cells were exposed with H_2_O_2_ for 6 h, and subsequently treated with melatonin, coenzyme Q10, and lecithin alone or in combination for further 24 h. Cell viability was assessed using the CCK‐8 assay. Eight‐week‐old male mice were intraperitoneally injected with D‐(+)‐galactose for 10 weeks and administrated with melatonin, coenzyme Q10, lecithin, or in combination for 5 weeks starting from the sixth week, followed by behavioral tests to assess the effectiveness in mitigating neurological deficits, and biochemical assays to explore the underlying mechanisms.

**Results:**

Exposure to H_2_O_2_ significantly reduced the viability of N2a cells and increased oxidative stress and tau phosphorylation, all of which were alleviated by treatment with melatonin, coenzyme Q10, lecithin alone, and, most noticeably, by combined treatment. Administration of mice with D‐(+)‐galactose‐induced oxidative stress and tau phosphorylation, brain aging, impairments in learning and memory, anxiety‐ and depression‐like behaviors, and such detrimental effects were mitigated by melatonin, coenzyme Q10, lecithin alone, and, most consistently, by combined treatment.

**Conclusions:**

These results suggest that targeting oxidative stress via supplementation of antioxidant nutrients, particularly in combination, is a better strategy to alleviate oxidative stress‐mediated neuronal loss and brain dysfunction due to age‐related neurodegenerative conditions.

## Graphical abstract

This study shows that compared with treatment with melatonin, coenzyme Q10, or lecithin alone, combined treatment results in more significant effects in reducing oxidative stress and tau protein phosphorylation, and alleviating oxidative stress‐mediated impairments in learning and memory.

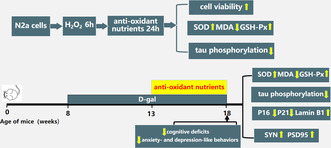

## 1 INTRODUCTION

Alzheimer's disease (AD) is a neurodegenerative disease affecting the brain with high incidence in the elderly.^1^, ^2^ The typical clinical manifestations include progressive impairment or loss of cognitive function.^3^ Senile plaques formed by extracellular β‐amyloid (Aβ) deposition,^4^ and neurofibrillary tangles formed by intracellular hyper‐phosphorylated tau protein aggregation in neuron^5^ are two hallmark pathological changes in the brains of AD patients, and they also represent key factors inducing or mediating cognitive damage. AD is the main cause of dementia, accounting for about 70% of the total cases of dementia. With the intensification of the aging of the world population, especially in the elderly over 75 years old, the incidence of AD increases year by year, and, as a matter of fact, has become a dire global public health problem. According to the World Alzheimer Report 2019, there are as many as 50 million AD patients worldwide and the number is expected to double every 20 years and increase to 152 millions by 2050, which not only brings heavy economic and emotional burdens to the society and families, but also poses a great challenge to the national medical and healthcare systems.^6^

It has been more than a century since AD was first reported, but there is still lack of effective measures to modify or prevent the disease progression, albeit drugs existing to alleviate the symptoms. Research efforts in the past decades have greatly increased the understanding of the clinical histopathology of AD, particularly neuronal cell death in different brain regions and impairments in cognitive and neurological functions.^7^ At the same time, with the multiple effects of genetic or environmental factors,^8^, ^9^ people are also increasingly aware of the extreme complexity of AD etiology.^10^ In terms of etiological classification, except for about 5% of familial hereditary AD cases, the rest are late‐onset or sporadic AD. Currently, the Aβ cascade hypothesis,^11^ tau protein hypothesis,^12^ and cholinergic hypothesis^13^ have been proposed for the pathogenesis of sporadic AD, but there is still no definitive conclusion on what mechanism dominates the occurrence and development of AD. Neuroinflammation,^14^ oxidative stress,^15^ mitochondrial dysfunction^16^ and autophagy lysosome system deficiency^17^ are considered to be the biological pathways that affect the pathology of AD. The disorder of any one pathway may trigger a vicious cycle of cell damage and neurodegeneration, which plays a crucial role in the development of AD. In particular, oxidative stress, which is closely related to AD and aging, can be targeted by antioxidants, which provides a possible direction for the treatment of AD.^18^

Reactive oxygen species (ROS) are a group of oxygen‐derived chemicals with short life but high activity. They are usually produced at a moderate level and eliminated in time through a variety of antioxidant mechanisms under physiological conditions. ROS as a signal molecule play an important role in maintaining cellular and tissue homeostasis.^19^ The increased production of free radicals can lead to oxidative stress, if the endogenous antioxidant capacity of the cell is insufficient to offset the production of these free radicals.^20^ The brain is vulnerable to oxidative stress due to its high oxygen consumption, relatively high level of polyunsaturated fatty acids that are sensitive to oxidation and relatively low level of antioxidant enzymes.^21^, ^22^ Oxidative stress is considered to be the main cause of AD progression.^23^ Severe neuronal oxidative damage have been observed in the atrophic brain tissues of AD patients.^24^ Chronic accumulation of AD‐related abnormal or misfolded proteins are well‐documented to induce excessive production of ROS, impair the antioxidant capacity of cell, or both, resulting in increased ROS levels and oxidative stress and thus changes in normal functions of cells in the brain.^25^

Aging is one of the main reasons for the gradual decline of brain functions, which can lead to progressive cognitive impairment. It is also one of the pathological risk factors of AD.^26^ During the process of aging, the increase in free radicals can damage the structure and function of the brain.^27^, ^28^ It was shown that the production of ROS is related to synaptic dysfunction and tau protein phosphorylation.^29^ The high level of ROS in the brain can result in the oxidation of proteins, lipids, and DNA, and eventually lead to neuronal loss and dysfunction.^30^, ^31^ Oxidative stress can also drive the progress of tau pathology in many ways, such as producing fatty acids, stimulating fibrosis tau protein aggregation, activating glycogen synthase kinase‐3 (GSK‐3β), and increasing hyperphosphorylation of tau proteins at multiple sites.^32^, ^33^ Under normal circumstances, endogenous antioxidants, such as trace elements (zinc and selenium), vitamins (vitamin C and vitamin E), polyphenols and coenzyme Q10, and antioxidant enzymes, including superoxide dismutase (SOD), catalase, and glutathione oxidoreductase, can resist or prevent oxidative damage. However, studies have reported that the level of antioxidant enzymes in the brains of patients with AD and mild cognitive impairment is significantly reduced, and the brain becomes more vulnerable to oxidative damage.^32^ Therefore, the use of antioxidants has been considered as a promising therapeutic strategy to alleviate oxidative stress‐induced or mediated neuronal damage and loss of function. Previous studies shown that long‐term administration of D‐(+)‐galactose in rodents can produce aging‐related changes that are similar to those observed in human beings, including the formation of excessive free radicals and the reduction of antioxidant enzyme activity. Therefore, D‐(+)‐galactose‐treated mice or rats have become a widely used experimental model for researching aging and underlying mechanisms.^34^, ^35^

Melatonin is a neurohormone secreted by the pineal gland of the brain that regulates the circadian rhythm and plays an important role in restricting neuroinflammation and oxidative damage.^35^, ^36^ Studies have shown that the melatonin level in AD patients is lower compared with that in normal subjects.^37^, ^38^ As shown in vivo and in vitro, melatonin supplementation effectively inhibited Aβ deposition and tau protein phosphorylation and, furthermore, treatment with melatonin restored cognitive function in AD transgenic mice.^39^, ^40^, ^41^ Mitochondria are the main source of ROS in cells and also the target for oxidative stress. Coenzyme Q10 is a vitamin‐like substance located in the inner membrane of mitochondria and acting as an electron carrier in the mitochondrial respiratory chain and has a redox capacity.^42^ Coenzyme Q10 is one of the most powerful endogenous membrane antioxidants and has been shown to protect neuronal cells from oxidative stress damage in vivo and in vitro.^43^ Moreover, coenzyme Q10 can reduce malondialdehyde (MDA) expression, regulate SOD activity, reduce Aβ plaque formation, protect AD‐related loss of synaptic plasticity and improve cognitive function.^44^, ^45^, ^46^ Lecithin is rich in choline and can combine with acetyl coenzyme A to form acetylcholine. Lecithin has been shown to reduce Aβ‐induced neuronal damage. Lecithin alone can also increase the expression of neurotrophic factors in the brain of mice, enhance the antioxidant capacity, and improve learning and memory.^47^ Therefore, the antioxidant therapy is considered to be an effective strategy to prevent or alleviate the pathological progress of oxidative neuronal damage and neurodegenerative conditions such as AD. The clinical trial results of using different nutritional supplements, singly or in combination in patients with AD, are different.^48^ As aforementioned, the pathogenesis of AD is highly complex, and the use of a single drug in preventing and treating AD is often not effective, leading to the notion that combined treatment with multiple drugs have a better or more significant effect.^49^, ^50^ Therefore, in this study, we selected melatonin, coenzyme Q10, and lecithin, three widely used nutrients with an antioxidant capacity, and examined the neuroprotective effects of applicating them individually or in combination on oxidative stress‐induced or mediated neuronal loss, and D‐(+)‐galactose‐induced neuronal damage and cognitive impairment, and the mechanisms of their actions.

## 2 MATERIALS AND METHODS

### 2.1 Chemicals, kits, and antibodies

D‐(+)‐galactose (G0750), hydrogen peroxide (H_2_O_2_) (323381), melatonin (M5250), and coenzyme Q10 (C9538) were purchased from Sigma‐Aldrich. Lecithin (L105732) was purchased from Aladdin. SOD assay kits (A001‐3‐2), MDA assay kits (A003‐1‐2), and glutathione peroxidase (GSH‐P_X_) assay kits (A005‐1‐2) were purchased from Nanjing Jiancheng Bioengineering Institute. Cell counting kit (CCK)‐8 (CK04) was purchased from Dojindo Molecular Technologies. Dimethyl sulfoxide (DMSO, 0219605580) was purchased from MP Biomedicals. Dulbecco's modified Eagle's medium (DMEM, SH30022.01B) was purchased from Hyclone. Fetal bovine serum (FBS, 16000–044) was purchased from Gibco. Penicillin–streptomycin (ST488) and horseradish peroxidase‐conjugated goat anti‐rabbit/mouse IgG (A0208, A0216) were purchased from Beyotime Biotechnology. Phenylmethylsulfonyl fluoride (36978) and AT8 antibody (Ser202/Thr205) (UB2696972) were purchased from Thermo Scientific. Anti‐β‐actin antibody (66009–1) was purchased from Proteintech. Antibodies for postsynaptic density 95 (PSD95) (2507), Lamin B1 (12586), Tau(Ser214) (77348), Tau(Thr231) (71429), GSK‐3β (12456), or p‐GSK‐3β(Ser9) (5558P) were purchased from Cell Signaling Technology. Antibodies for Tau5 (ab80579) and synaptophysin (ab32127) were purchased from Abcam. Antibodies for Tau(Ser396) (13400), Tau(Ser356) (11101), Tau(Ser404) (11112) or Tau(Thr205) (11108) were purchased from Signalway Antibody. Antibodies for P16 (SC‐1661) and P21 (SC‐6246) were purchased from Santa Cruz.

### 2.2 Cell culture and treatments

Mouse neuroblastoma Neuro‐2a (N2a) cells used in the study were purchased from the cell bank of the Chinese Academy of Sciences. Cells were maintained in DMEM medium supplemented with 10% FBS, penicillin at 100 units/mL and streptomycin at 100 mg/mL at 37°C in a humid tissue culture incubator with 5% CO_2_. For experiments, cells were seeded in 6‐well plates at a density of 2.5 × 10^5^ per well and cultured for 24 h before use. In some experiments, cells were exposed to H_2_O_2_ alone at indicated concentrations for 24 h. In experiments testing the effects of melatonin, coenzyme Q10 and lecithin individually or in combination, cells were exposed to 250 μM H_2_O_2_ for 6 h and cultured in media containing 10 μM melatonin, 25 μM coenzyme Q10, 10 μM lecithin, or all three (combined treatment) for further 24 h. Cells treated in combination without prior exposure to H_2_O_2_ were used as negative controls.

### 2.3 Mice and treatments

C57/BL6 male mice (6–8 weeks and 20–25 g) used in this study were purchased from SPF (Beijing) Biotechnology. A total of 84 mice were used. Mice were housed in standard cages with up to six mice per cage, with temperature and humidity set at 23 ± 2°C and 60 ± 5%, respectively, and light–dark cycle for 12 h each session, and had free access to water and food. After adaptation for 1 week, mice were randomly divided into the following seven groups, with 12 mice in each group: control, D‐(+)‐galactose, D‐(+)‐galactose plus melatonin, D‐(+)‐galactose plus coenzyme Q10, D‐(+)‐galactose plus lecithin, D‐(+)‐galactose plus combined treatment, and control plus combined treatment. Mice were intraperitoneally injected with D‐(+)‐galactose (120 mg/kg/d), or an equal volume of sterile normal saline (0.9% NaCl) for 10 consecutive weeks. At the beginning of the sixth week of the experiment, melatonin (10 mg/kg), coenzyme Q10 (200 mg/kg), lecithin (60 mg/kg), and combination of all three were simultaneously given by gavage, once a day, starting from the first day of the sixth week until the end of the tenth week.^22^, ^51^, ^52^

### 2.4 Western blotting

Proteins were isolated from brain tissues or cells using RIPA lysis buffer containing phosphatase inhibitor cocktail and protease inhibitor phenylmethanesulfonylfluoride. Protein concentrations were detected using the BCA method and 10–15 μg proteins were separated by electrophoresis in 10%–15% sodium dodecyl sulfate–polyacrylamide gel electrophoresis gels, and then transferred to nitrocellulose membranes. The membranes were blocked in PBS containing 5% skim milk at room temperature for 1 h and incubated with the primary antibody at 4°C overnight. After washed in PBS, the membranes were incubated with the secondary antibody at room temperature for 2 h. All the antibodies were used at a dilution of 1: 1000. Protein bands were visualized using an enhanced chemiluminescence kit, and quantified using ImageJ software.

### 2.5 Behavioral testing

#### 2.5.1 Morris water maze test

The Morris water maze test was performed as described previously.^53^ A circular pool was filled with opaque water, and a hidden platform was placed below the surface of the water in one of the quadrants. The movement trajectory of mice was recorded using a digital tracking device. Briefly, the mice were trained to find the hidden platform for five consecutive days with four trials per day. On the seventh day, the platform was removed while other experimental conditions remained the same. The mice were gently lowered from the opposite quadrant of the platform into the water, allowing them to explore freely in the water for 2 min. The swimming speed, movement trajectory, escape latency, shuttle number of times, and dwell time on the platform were recorded for each mouse, and the spatial memory ability was analyzed.

#### 2.5.2 Y‐maze test

The Y‐maze test was also used to evaluate spatial learning and memory especially hippocampus‐dependent memory.^54^ During training, each mouse was allowed to freely explore within the open arm for 10 min. A video camera was placed above the maze to record the movements of the mice. The spatial memory of mice was tested at 1 h and 24 h after training. In the detection phase, the barrier was removed to allow the mouse to freely explore for 5 min, while the residence time and the number of shuttles in the novel arm were recorded. The spatial recognition ability was evaluated by the ratio of their residence time in the novel arm to total time within 5 min.

#### 2.5.3 Open field test

The open field test was used to evaluate autonomous behaviors, exploring behaviors, and tension of animals in new and different environments. The mice were put into the testing room 1 day in advance to adapt to the environments. During the test, the mice were placed in the box to explore freely for 5 min, and the residence times in the central and peripheral regions were recorded.

#### 2.5.4 Elevated plus maze test

The elevated plus maze test is one of the most widely used tests to measure anxiety‐like behaviors. The test is based on the natural aversion for open and elevated areas, as well as on their natural spontaneous exploratory behaviors in novel environments.^55^ The day before the test, the mice were brought to the testing room in advance to familiarize with the environments. During the test, the mice were placed in the central area facing the closed arm and allowed to explore freely for 5 min. Meanwhile, the residence time and shuttle time for each mouse in the open and closed arms were recorded. The ratio of open arm dwell time to closed arm dwell time was used as an indicator of anxiety‐like behaviors.

### 2.6 SOD, MDA, and GSH‐Px analysis

N2a cells and brain tissues were harvested, and the concentrations of SOD, MDA, and GSH‐Px were measured using commercial assay kits according to the manufacturer's instructions.

### 2.7 Statistical analysis

All data were presented as mean ± standard deviation (SD) and analyzed using GraphPad Prism software (version 6). The data were tested for normality using the Shapiro–Wilk test. Student's *t*‐test was used for the comparison between two groups, and one‐way analysis of variance analysis (ANOVA) followed by Tukey's test for the comparison of multiple groups, with *p* < 0.05 considered to have a statistically significant difference.

## 3 RESULTS

Treatments with antioxidant nutrients mitigate H_2_O_2_‐induced oxidative stress and neuronal death in N2a cells.

**FIGURE 1:**
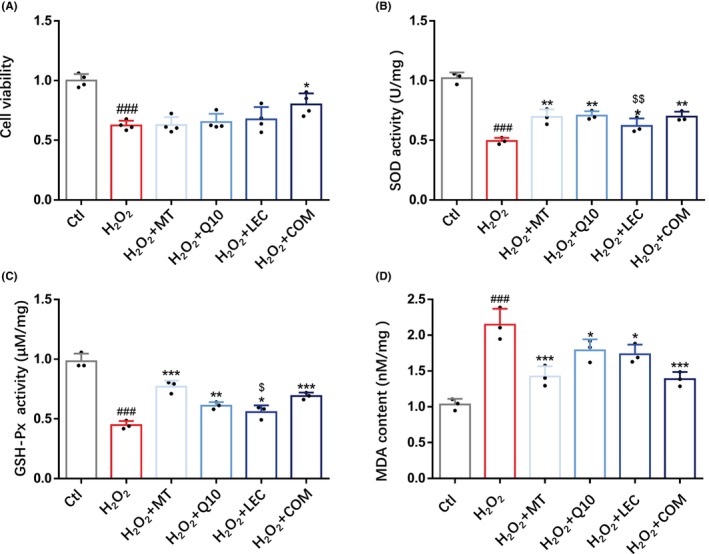
Treatments with antioxidant nutrients improve the viability and reduce oxidative stress in H_2_O_2_‐induced N2a cells. (A) The effects of treatment with MT, Q10, LEC, and COM for 24 h on the viability of N2a cells exposed to H_2_O_2_ for 6 h prior to treatment. (B–D) The activities of SOD and GSH‐P_X_ and content of MDA in indicated groups. Data are expressed as mean ± SD; *n* = 3–4 for each group. ^###^ *p* < 0.005 vs Ctl; *p < 0.05, **p < 0.01 and ***p < 0.005 versus H_2_O_2_; ^$^ *p* < 0.05 and ^$$^ *p* < 0.01 versus H_2_O_2_ + COM.

To compare the effectiveness of treatments with melatonin, coenzyme Q10, lecithin individually and in combination in protecting against oxidative stress‐induced neuronal damage, we firstly used H_2_O_2_‐induced death of N2a cells as an in vitro model of oxidative stress‐induced neuronal death. Exposure of N2a cells to 0, 125, 250, 500, and 1000 μM H_2_O_2_ for 6 h concentration‐dependently reduced cell viability (Figure S1A), assessed using the CCK‐8 assay. The cell viability was 63.3 ± 1.6% after exposure to 250 μM H_2_O_2_, which was used in further studies. Treatments of N2a cells, following exposure to H_2_O_2_, with melatonin, coenzyme Q10, and lecithin in combination significantly reduced H_2_O_2_‐induced cell death and, by contrast, treatment with melatonin, coenzyme Q10, or lecithin individually was ineffective (Figure 1A). To confirm that exposure to H_2_O_2_ indeed induced oxidative stress, we analyzed the activities of SOD and GSH‐P_X_, two enzymes important in equipping cells with an antioxidant capacity,^56^ and MDA, an end‐product of lipid peroxidation widely used as a biomarker to measure oxidative stress,^57^ in N2a cells. As previously reported,^58^ exposure to H_2_O_2_ led to a significant decrease in the activity levels of SOD and GSH‐P_X_ (Figure 1B,C) and a significant increase in the level of MDA (Figure 1D), both of which were significantly attenuated by treatment with melatonin, coenzyme Q10 and lecithin individually and in combination (Figure 1B–D). Of notice, combined treatment overall was more consistent and effective in mitigating H_2_O_2_‐induced decrease in the activity levels of SOD and increase in the level of MDA (Figure 1B,D). Neither the cell viability nor the activity of SOD was altered by combined treatment in N2a cells without prior exposure to H_2_O_2_ (Figure S1B). Taken together, these results provide the first indication that treatment with melatonin, coenzyme Q10, and lecithin in combination is more effective than treatment with individual nutrient in mitigating oxidative stress and oxidative stress‐induced neuronal death.

### 3.1 Treatments with antioxidant nutrients reduce H_2_O_2_ ‐induced tau phosphorylation and GSK‐3β activation in N2a cells

**FIGURE 2:**
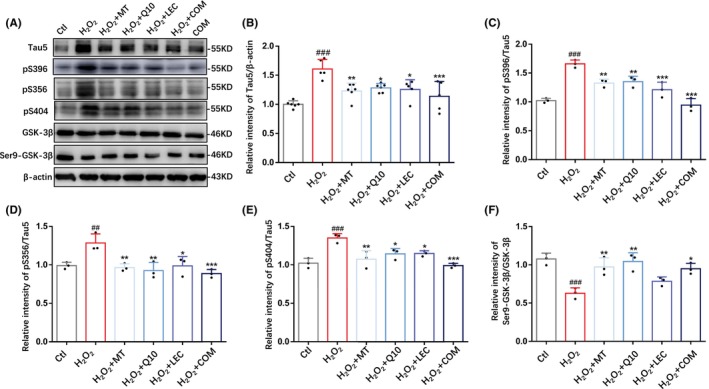
Treatments with antioxidant nutrients reduce tau protein expression and phosphorylation levels, and GSK‐3β activity in H_2_O_2_‐exposed N2a cells. (A) Representative western blots showing Tau5, p‐Tau(Ser396), p‐Tau(Ser356), p‐Tau(Ser404), GSK‐3β, Ser9‐GSK‐3β and β‐actin in indicated groups. (B‐F) Summary of Tau5/β‐actin, p‐Tau(Ser396)/β‐actin, p‐Tau(Ser356)/β‐actin, p‐Tau(Ser404)/β‐actin, Ser9‐GSK‐3β/GSK‐3β. Data were expressed as mean ± SD; *n* = 3–6 for each group. ^##^ *p* < 0.01 and ^###^ *p* < 0.005 vs Ctl; *p < 0.05, **p < 0.01, and ***p < 0.005 vs H_2_O_2_.

Oxidative stress can aggravate the phosphorylation of tau proteins in neuronal cells, which is an important pathological factor inducing neurodegenerative diseases including AD^30^ Therefore, we next examined the expression and phosphorylation of tau proteins in N2a cells after exposure to H_2_O_2_ and, furthermore, compared the effectiveness of treatments with melatonin, coenzyme Q10, lecithin, or in combination. As shown by western blotting (Figure 2A), exposure to H_2_O_2_ significantly increased the levels of tau protein expression (Tau5) (Figure 2B) and the phosphorylation levels at Ser396, Ser356, and Ser404 (Figure 2C–E).^59^ Such effects were reversed to varying degree by treatment with melatonin, coenzyme Q10 or lecithin. Of notice, H_2_O_2_‐induced tau phosphorylation was more consistently and effectively suppressed by combined treatment, albeit there was no statistically significant difference compared to treatment with melatonin, coenzyme Q10, or lecithin alone (Figure 2A–E). GSK‐3β activation by phosphorylation at Ser9 is important in promoting tau protein phosphorylation, particularly in the context of AD.^60^ Exposure to H_2_O_2_ decreased the p‐GSK‐3β (Ser9)/GSK‐3β ratio (Figure 2F) without altering GSK‐3β protein expression (Figure S2A), indicating that oxidative stress activates GSK‐3β, as reported previously.^61^ H_2_O_2_‐induced GSK‐3β activation was attenuated by combined treatment, as well as treatment with melatonin, coenzyme Q10, but not lecithin (Figure 2F). Neither the levels of tau protein expression and phosphorylation nor the GSK‐3β activity (Figure S2B) in N2a cells under control conditions were changed by combined treatment. Collectively, these results show that treatments with melatonin, coenzyme Q10, and lecithin, particularly combined treatment, are effective in reducing oxidative stress‐induced tau phosphorylation and GSK‐3β activation in neuronal cells.

### 3.2 Treatments with antioxidant nutrients alleviate D‐(+)‐galactose‐induced oxidative stress, tau phosphorylation, and GSK‐3β activation in mice

**FIGURE 3:**
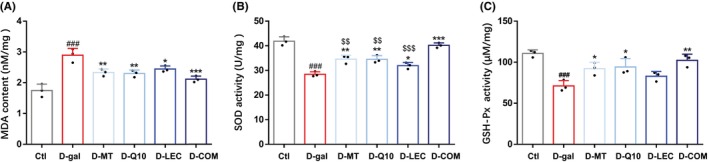
Treatments with antioxidant nutrients reduce D‐(+)‐galactose‐induced oxidative stress in mice. (A–C) The contents of MDA and the activities of SOD and GSH‐P_X_ in indicated groups of mice. Data are expressed as mean ± SD; n = 3 for each group. ^**###**^ *p* < 0.005 versus Ctl, *p < 0.05, **p < 0.01 and ***p < 0.005 versus D‐gal; ^$$^ *p* < 0.01, ^$$$^ *p* < 0.005 versus D‐COM.

To evaluate the effectiveness of antioxidant nutrients in protecting against oxidative stress‐related neurological deficits in vivo, we examined the effectiveness of treatments with melatonin, coenzyme Q10, lecithin, or in combination in mice intraperitoneally injected with D‐(+)‐galactose, which as has been well‐documented can induce oxidative stress and neurological deficits associated with neurodegenerative diseases like AD.^62^ Injection of D‐(+)‐galactose led to a significant decrease in the activities of SOD and GSH‐P_X_ and an increase in the level of MDA in the brain, which were significantly alleviated by treatment with melatonin, coenzyme Q10, lecithin, or in combination (Figure 3A–C), except that D‐galactose‐induced decrease in the GSH‐Px activity was not increased by treatment with lecithin (Figure 3C). The levels of SOD, GSH‐Px, and MDA were not altered by combined treatment in control mice (i.e., injection with 0.9% NaCl) (Figure S3).

**FIGURE 4:**
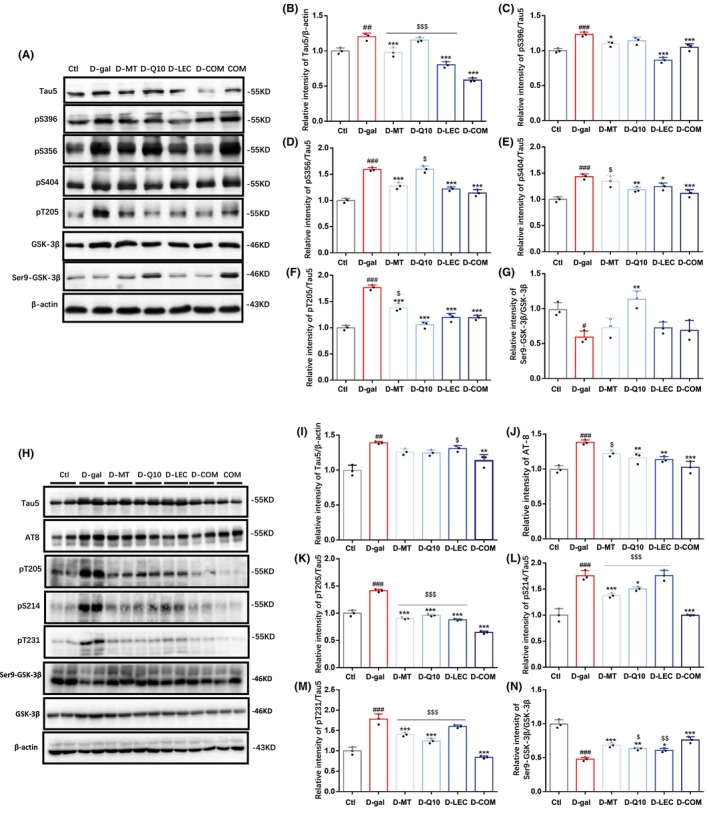
Treatments with antioxidant nutrients reduce D‐(+)‐galactose‐induced tau phosphorylation and GSK‐3β activation in mice. (A) Representative western blots showing Tau5, p‐Tau(Ser396), p‐Tau(Ser356), p‐Tau(Ser404), p‐Tau(Thr205), Ser9‐GSK‐3β, GSK‐3β and β‐actin in the cortex. (B‐G) Summary of Tau5/β‐actin, p‐Tau(Ser396)/β‐actin, p‐Tau(Ser356)/β‐actin, p‐Tau(Ser404)/β‐actin, p‐tau(Thr205)/β‐actin and Ser9‐GSK‐3β/GSK‐3β in the cortex. (H) Representative western blots showing Tau5, AT8, p‐Tau(Thr205), p‐Tau(Ser214), p‐Tau(Thr231), Ser9‐GSK‐3β, GSK‐3β and β‐actin in the hippocampus. (I‐N) Summary of Tau5/β‐actin, AT8/β‐actin, p‐Tau(Thr205)/β‐actin, p‐Tau(Ser214)/β‐actin, p‐Tau(Thr231)/β‐actin and Ser9‐GSK‐3β/GSK‐3β in the hippocampus. Data are expressed as mean ± SD; *n* = 3 for each group. ^#^ *p* < 0.05, ^##^ *p* < 0.01 and ^###^ *p* < 0.005 vs Ctl; *p < 0.05, **p < 0.01 and ***p < 0.005 vs D‐gal; ^$^ *p* < 0.05, ^$$^ *p* < 0.01, and ^$$$^ *p* < 0.005 versus D‐COM.

We also examined the expression and phosphorylation of tau proteins and the activity of GSK‐3β in the brain of D‐(+)‐galactose‐injected mice (Figure 4A,H). Injection of D‐(+)‐galactose upregulated tau protein expression in both cortex and hippocampus (Figure 4B,I), and increased the levels of phosphorylation at multiple sites including Ser396, Ser356, Ser404, and Thr205 in the cortex (Figure 4C–F) and Ser202/Thr205, Thr205, Ser214, and Thr231 in the hippocampus (Figure 4J–M). D‐(+)‐galactose‐induced phosphorylation of tau proteins was consistently alleviated by combined treatment, while treatment with individual nutrient appeared less effective or, in some cases, ineffective, for example, Ser404 and Thr205 in the cortex and Ser202/Thr205 in the hippocampus (melatonin), Ser356 in the cortex (coenzyme Q10), Tau5 in the hippocampus (lecithin) (Figure 4D–F, I,J). Combined treatment resulted in a greater inhibition of Tau5 in the cortex (Figure 4B), and phosphorylation at Thr205, Ser214 and Thr231 in the hippocampus (Figure 4K–M). Injection of D‐(+)‐galactose also enhanced the activity of GSK‐3β, indicated by a significant decrease in the pS9‐GSK‐3β/GSK‐3β in the cortex and hippocampus. D‐(+)‐galactose‐induced GSK‐3β activation in the cortex was reversed by treatment with coenzyme Q10, but not with melatonin, lecithin or in combination, and D‐(+)‐galactose‐induced GSK‐3β activation in the hippocampus was significantly reduced by treatment with melatonin, coenzyme Q10, lecithin and combination (Figure 4G,N). The expression and phosphorylation of tau proteins and the activity of GSK‐3β in control mice were not altered by combined treatment, except a modest but significant reduction in the levels of tau5 and phosphorylation at Ser396 and Ser404 in the cortex, phosphorylation at Thr205 and Ser214, and pS9‐GSK‐3β/GSK‐3β in the hippocampus (Figure S4). These results from in vivo experiments are highly consistent with those from in vitro experiments, providing further evidence to support the notion that treatments with antioxidant nutrients, particularly combined treatment, are effective in alleviating D‐(+)‐galactose‐induced oxidative stress, tau protein phosphorylation, and GSK‐3β activation in the brain of mice.

### 3.3 Treatments with antioxidant nutrients alleviate D‐(+)‐galactose‐induced brain aging in mice

**FIGURE 5:**
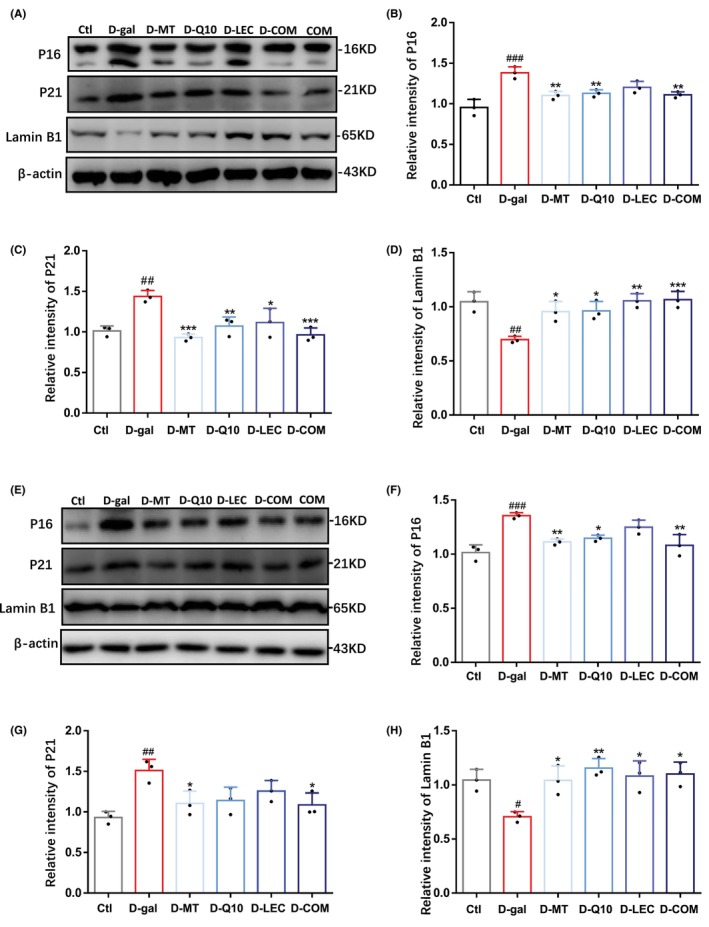
Treatments with antioxidant nutrients reverse D‐(+)‐galactose‐induced brain aging. (A–D) Representative western blots and summary of P16, P21, and Lamin B1 in the cortex. (E–H) Representative western blots and summary of P16, P21, and Lamin B1 in the hippocampus. Data are expressed as mean ± SD; *n* = 3 for each group. ^#^ *p* < 0.05, ^##^ *p* < 0.01 and ^###^ *p* < 0.005 vs Ctl; *p < 0.05, **p < 0.01, and ***p < 0.005 vs D‐gal.

Oxidative stress is known for its ability to activate the senescence‐related signaling pathways to exacerbate cellular senescence that is accompanied by cell cycle arrest.^63^ Therefore, we examined the effectiveness of treatments with melatonin, coenzyme Q10, lecithin, or in combination on the levels of P16 and P21, two key cell cycle inhibitors, and Lamin B1, a nuclear marker.^64^ Injection of D‐(+)‐galactose resulted in a significant increase in the levels of P16 and P21 and a significant decrease in the expression level of Lamin B1 in both cortex and hippocampus (Figure 5A,E). In the cortex and hippocampus, D‐(+)‐galactose‐induced increase in the expression of P16 and P21 was prevented by combined treatment, and D‐(+)‐galactose‐induced increase in the expression of P21 was also attenuated by treatment with melatonin, coenzyme, or lecithin in the cortex and by treatment with melatonin in the hippocampus, whereas D‐(+)‐galactose‐induced increase in the expression of P16 was mitigated by treatment with melatonin or coenzyme Q10, but not lecithin (Figure 5B,C,F,G). D‐(+)‐galactose also induced a decrease in the expression level of Lamin B1 in the cortex and hippocampus, which was significantly reversed by treatment with melatonin, coenzyme Q10, lecithin, or in combination (Figure 5D,H). The expression of P16, P21, and Lamin B1 were not altered by combined treatment in control mice (Figure S5). These results indicate that treatments with antioxidant nutrients, particularly in combination, are effective in alleviating D‐(+)‐galactose‐induced senescence leading to brain aging.

### 3.4 Treatments with antioxidant nutrients prevent D‐(+)‐galactose‐induced synaptic loss in mice

**FIGURE 6:**
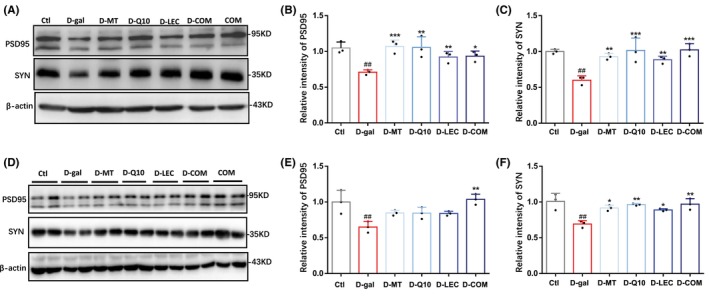
Treatments with antioxidant nutrients improve D‐(+)‐galactose‐induced reduction in the levels of synaptic proteins in mice. (A–C) Representative western blots and summary of PSD95 and synaptophysin (SYN) in the cortex. (D–F) Representative western blots and summary of PSD95 and SYN in the hippocampus. Data are expressed as mean ± SD; *n* = 3 for each group. ^##^ *p* < 0.01 vs Ctl; *p < 0.05, **p < 0.01, and ***p < 0.005 vs D‐gal.

The integrity and plasticity of synapses in the brain are vital for the transmission of information and also necessary for learning and memory. Studies have shown that oxidative stress causes synaptic loss, thereby impairing neurotransmission and cognitive functions. Therefore, we further examined the effectiveness of treatments with melatonin, coenzyme Q10, lecithin, or in combination on the expression levels of PSD95 and synaptophysin proteins, which reflect the number of synapses. Injection of D‐(+)‐galactose reduced the expression levels of both PSD95 and synaptophysin proteins in the hippocampus, indicating loss of synapses. D‐(+)‐galactose‐induced reduction in the expression levels of PSD95 was reversed by combined treatment, but not by treatment with melatonin, coenzyme Q10 or lecithin in the hippocampus (Figure 6A,B,D,E). D‐(+)‐galactose‐induced reduction in the expression level of synaptophysin expression was alleviated by treatment with melatonin, coenzyme Q10, lecithin, and in combination (Figure 6A,C,D,F). The expression of PSD95 and synaptophysin proteins in control mice was not altered by combined treatment except the expression of PSD95 in the hippocampus (Figure S5). These results demonstrate that treatments with melatonin, coenzyme Q10, or lecithin, particularly in combination, are effective in protecting oxidative stress‐induced synaptic loss in the brain.

### 3.5 Treatments with antioxidant nutrients improve D‐(+)‐galactose‐induced cognitive deficits in mice

**FIGURE 7:**
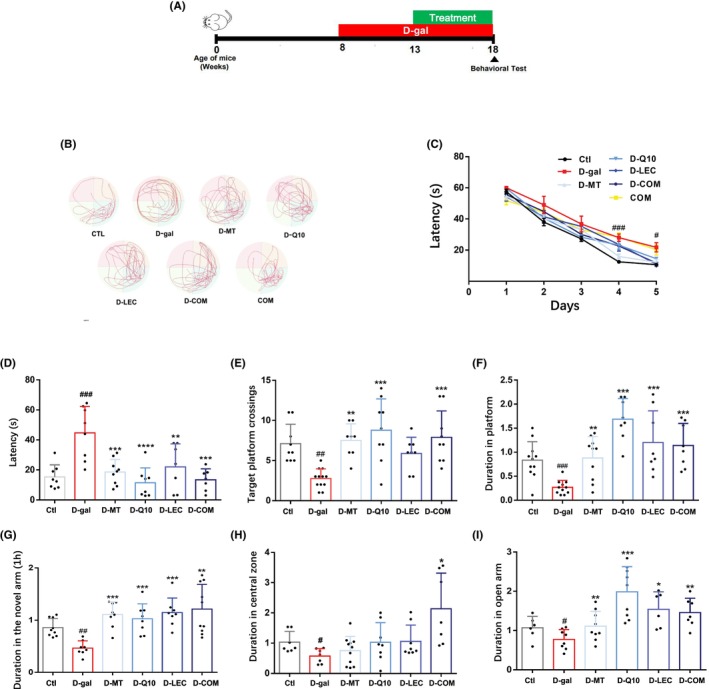
Treatments with antioxidant nutrients improve D‐(+)‐galactose‐induced cognitive deficits in mice. (A) Timeline of animal modeling, testing and treatment. (B) The swimming trajectory map detected by Morris water maze (MWM) test on the seventh day. (C) Escape latency during MWM training. (D) The platform escape latency detected by MWM on the seventh day. (E) The number of platform shuttles detected by MWM on the seventh day. (F) The percentage of the platform dwell time detected by MWM on the seventh day. (G) The percentage of time spent in the novel arm at 1 h detected by Y‐maze test. (H) The percentage of time mice spent in the central area detected by open field experiment. (I) The percentage of time spent in the open arm detected by elevated plus maze. Data are expressed as mean ± SD; *n* = 6–12 for each group. ^#^ *p* < 0.05, ^##^ *p* < 0.01 and ^###^ *p* < 0.005 vs Ctl; *p < 0.05, **p < 0.01, and ***p < 0.005 versus D‐gal.

It is known that long‐term injection of D‐(+)‐galactose can cause symptoms bearing similarity to those in AD.^65^ Therefore, we evaluated the effectiveness of treatments with melatonin, coenzyme Q10, lecithin, or in combination on oxidative stress‐induced cognitive deficits using the Morris water maze test and Y‐maze test (Figure 7A). Administration of D‐(+)‐galactose or these antioxidant nutrients alone or together was without significant effect on the body weight, growth, or swimming speed of the mice (Figure S6A,B). As previously reported,^66^ injection of D‐(+)‐galactose led to impairments in learning, indicated by a longer escape latency during the training session in Morris water maze testing (Figure 7B,C) and, furthermore, in memory, indicated by increased latency, reduced target platform crossing times and shortened dwell time in the platform area (Figure 7D–F). D‐(+)‐galactose‐induced impairments in both learning and memory were largely prevented by treatment with melatonin, coenzyme Q10, lecithin, or in combination (Figure 7D–F). Similarly, as shown in Y‐maze testing, injection of D‐(+)‐galactose impaired short‐term spatial memory, indicated by shortened duration of residing in the novel forearm and such impairment was prevented by treatment with melatonin, coenzyme Q10, lectin, and in combination (Figure 7G). The long‐term spatial memory was significantly improved only by combined treatment (Figure S6C). The learning and memory in control mice was not improved by combined treatment (Figure S6D). These results show that D‐(+)‐galactose‐induced oxidative stress‐mediated impairments in learning and memory in mice can be reversed or improved by treatment with melatonin, coenzyme Q10, and lecithin and, more consistently and effectively, by combined treatment.

### 3.6 Treatments with antioxidant nutrients attenuate D‐(+)‐galactose‐induced anxiety‐ and depression‐like behaviors in mice

There is increasing evidence to suggest individuals with AD and other neurodegenerative conditions are prone to experience anxiety and depression.^67^ Therefore, we finally determined the effectiveness of treatments with melatonin, coenzyme Q10, lecithin, or in combination on anxiety‐ and depression‐like behaviors in D‐(+)‐galactose‐injected mice, using the open field test and elevated plus maze test. Injection of D‐(+)‐galactose‐induced anxiety‐like and depression‐liked behaviors, manifested by a decrease in the ratio of time spent in the central area to total time in the open field test (Figure 7H) and a decrease in the percentage of open arm residence time in the elevated plus maze test (Figure 7I). D‐(+)‐galactose‐induced anxiety‐ and depression‐like behaviors were also significantly reduced by treatment with melatonin, coenzyme Q10 or lecithin in the plus maze test, but not in the open field test (Figure 7H,I). There was no change in the behaviors of control mice after combined treatment (Figure S6D). Collectively, these results indicate that treatment combining melatonin, coenzyme Q10 and lecithin is more consistent and effective than treatment with individual nutrients in improving D‐(+)‐galactose‐induced anxiety‐ and depression‐like behaviors.

## 4 DISCUSSION

Oxidative damage has been well‐documented in the brains of aging‐related neurodegenerative conditions such as AD, and thus antioxidant is considered as a potential strategy of treating AD. In this study, we present consistent evidence from in vitro and in vivo experiments to show that treatments with antioxidants, melatonin, coenzyme Q10, and lecithin, particularly in combination of them, can effectively protect oxidative stress‐induced neuronal loss and oxidative stress‐mediated neurological dysfunction.

Hippocampus is essential for consolidating short‐term memory into long‐term memory and forming spatial memory, but it is extremely vulnerable to oxidative stress. As reported by previous studies, long‐term intraperitoneal injection of D‐(+)‐galactose in mice impaired spatial learning and memory^68^, ^69^, ^70^ and, in addition, induced anxiety‐ and depression‐like behaviors.^71^, ^72^ Treatment of AD mice with melatonin, coenzyme Q10, or lecithin improved learning and memory through various mechanisms.^73^, ^74^, ^75^ However, previous studies mainly focused on the effects of treatment with individual nutrient. In this study, examined the effects of treatment with melatonin, coenzyme Q10, and lecithin alone and, more importantly, compared the effects of treatment combining three nutrients. Overall, combined treatment was more consistent and significant in protecting against oxidative stress‐induced neuronal death in vitro, and alleviating D‐(+)‐galactose‐induced oxidative stress‐mediated impairments in spatial learning and memory, and anxiety‐like behaviors in mice. It is known that many markers of oxidative stress including MDA were elevated and the levels of antioxidant enzymes such as SOD and GSH‐Px were decreased in the brains of AD patients or animal models.^76^, ^77^, ^78^ Consistently, this study showed that the MDA level was significantly increased and the activities of SOD and GSH‐Px reduced in N2a cells exposed to H_2_O_2_ and in the brain tissues of mice injected with D‐(+)‐galactose, all of which were attenuated to various degree by treatments with melatonin, coenzyme Q10, or lecithin and, more significantly, by combined treatment, indicating a stronger antioxidant effect of combined treatment.

In the present study, we also showed that long‐term administration of D‐(+)‐galactose in mice led to increased GSK‐3β activity in the hippocampus and increased phosphorylation of tau proteins at multiple sites (Ser396, Ser356, and Thr205), similar to the results of previous studies.^79^, ^80^, ^81^ It was previously reported that treatment with melatonin alleviated endoplasmic reticulum stress by down‐regulating the GSK‐3β activity and protected against kainic acid‐induced neurodegeneration and tau protein hyperphosphorylation, and that treatment with coenzyme Q10 alleviated sevoflurane‐induced neuroinflammation by regulating the APOE expression and phosphorylation of tau proteins in mouse hippocampal neurons.^82^, ^83^, ^84^ Our results presented in this study showed that D‐(+)‐galactose‐induced GSK‐3β activity in cortical tissues was only significantly inhibited by treatment with coenzyme Q10, whereas D‐(+)‐galactose‐induced GSK‐3β activity in the hippocampus was inhibited by treatment with melatonin, coenzyme Q10 or lecithin, and the inhibition by combined treatment was more consistent and significant. In addition, the increase in tau protein expression in the hippocampus was significantly reduced by combined treatment, but not by treatment with individual nutrient. Likewise, treatment with individual nutrient reduced the phosphorylation levels of tau proteins at Ser356 and Thr205 in both hippocampus and cortex, the phosphorylation levels of tau proteins at multiple sits were effectively reduced by combined treatment. Collectively, these results suggest that combined treatment can significantly enhance the antioxidant capacity, thereby reducing D‐(+)‐galactose‐induced oxidative stress and tau protein phosphorylation. As proposed by a previous study,^84^ there exists a vicious circle between tau pathological changes and oxidative stress. Thus, it is worth mentioning that D‐(+)‐galactose‐induced hyperphosphorylation of tau proteins is not only a consequence of GSK‐3β activation but also one cause of oxidative stress.

Synapse has a high energy demand and contains a large number of mitochondria, and represents a target vulnerable to oxidative stress. It was showed in D‐(+)‐galactose‐treated rats that the synaptic density in the forebrain was significantly reduced and cholinergic neurons were lost.^85^ In this study, we examined the levels of synaptophysin and PSD95, core components of the synaptic structure,^86^, ^87^ in mouse cortex and hippocampus. Injection of D‐(+)‐galactose significantly reduced the levels of synaptophysin and PSD95 proteins in the hippocampus, indicating synaptic damage. Treatment with melatonin, coenzyme Q10, lecithin alone or in combination significantly mitigated D‐(+)‐galactose‐induced loss of synaptophysin and PSD95 in the hippocampus. Moreover, combined treatment was more significant in restoring the levels of synaptophysin and PSD95 in the hippocampus compared to treatment with individual nutrient. These results suggest that combined treatment is more effective and significant in protecting oxidative stress‐induced synaptic damage and improving impairments in learning and memory in D‐(+)‐galactose‐administrated mice.

## 5 CONCLUSION

In conclusion, this study provides evidence from in vitro and in vivo experiments to show that compared with treatment with melatonin, coenzyme Q10, or lecithin alone, combined treatment results in more consistent, effective and significant effects in reducing oxidative stress and tau protein phosphorylation, and alleviating oxidative stress‐mediated impairments in learning and memory. Further researches are required to gain an in‐depth understanding of the mechanisms of actions. Nonetheless, our finding suggests that combined use of antioxidant nutrients is a better strategy to reduce oxidative damage and treat AD.

## AUTHOR CONTRIBUTIONS

WL, WYJ, SZZ, TWY, LH, and YYL performed the research. WL and WJ‐Z designed the research. WL and WYJ analyzed the data. WL, WYJ, and JL‐H wrote the manuscript. WL, JL‐H, and WJ‐Z revised paper. All authors read and approved the final manuscript.

## FUNDING INFORMATION

This study was supported by the Key Research and Development Project of Henan Province (NO. 232102311035) and Henan Key Laboratory of Neurorestoratology (NO. HNSJXF‐2021‐012) and the Open Project Program of the Third Affiliated Hospital of Xinxiang Medical University (No. KFKTYB202121).

## CONFLICT OF INTEREST STATEMENT

All authors declare no conflict of interest.

## Supporting information


Figure S1.

Figure S2.

Figure S3.

Figure S4.

Figure S5.

Figure S6.

## Data Availability

The authors confirm that the data supporting the findings of this study are available within the article and/or its supplementary materials.
