# Supplementary figures and images for: Effectiveness and mechanisms of combined use of antioxidant nutrients in protecting against oxidative stress‐induced neuronal loss and related neurological deficits
Source: CNS Neurosci Ther. 2024 Jul 28;30(7):e14886. doi: 10.1111/cns.14886 (PMC11284237; doi:10.1111/cns.14886)

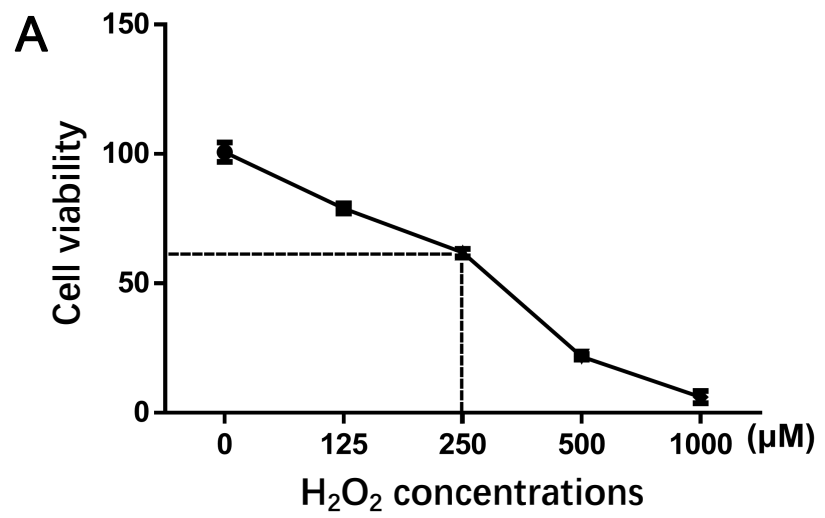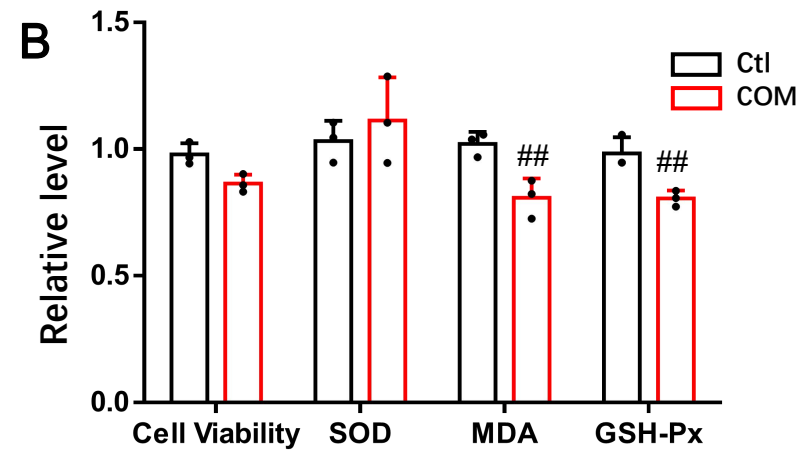

Supplement: Supplementary file 1 — Figure S1. [file CNS-30-e14886-s002.pdf]

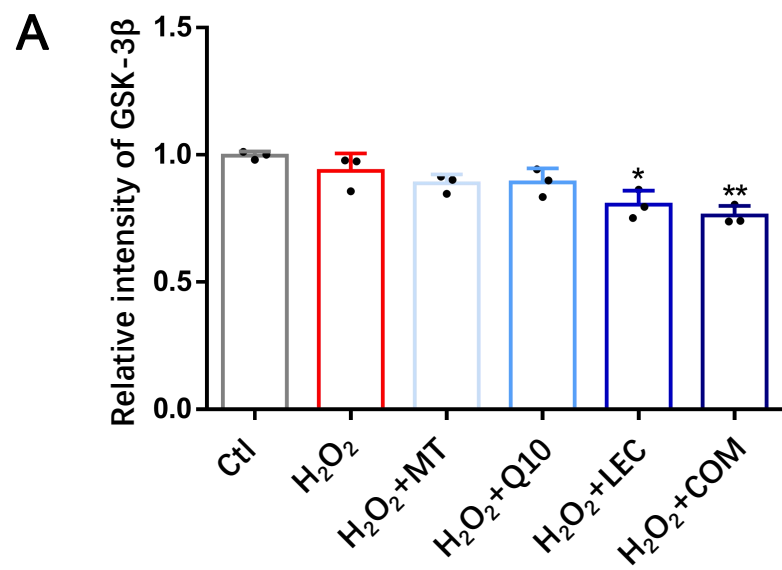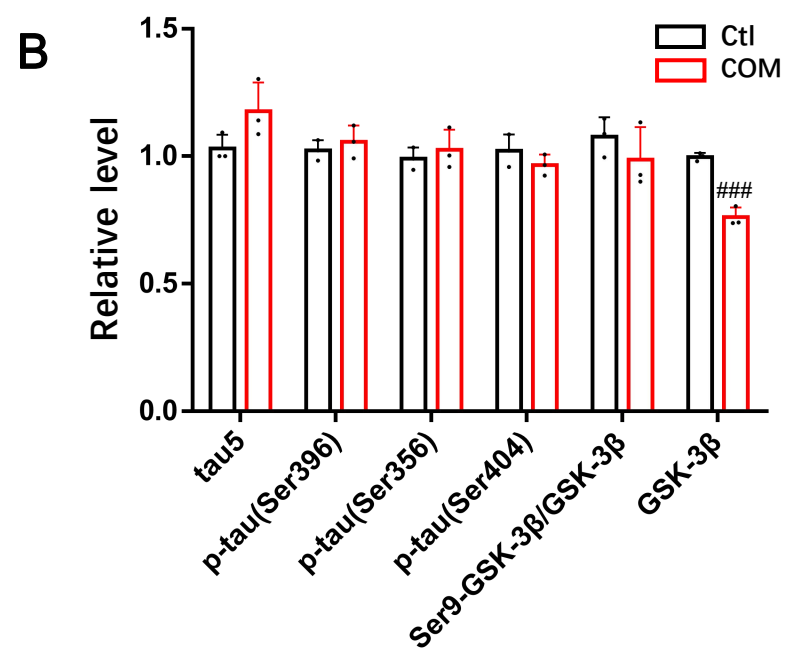

Supplement: Supplementary file 2 — Figure S2. [file CNS-30-e14886-s006.pdf]

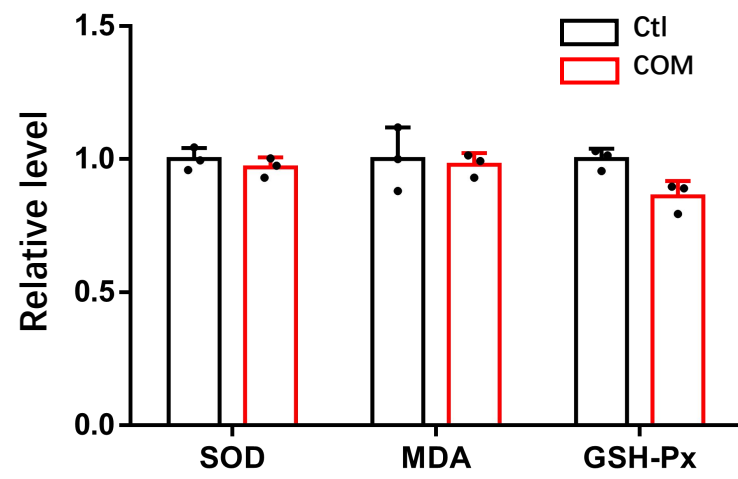

Supplement: Supplementary file 3 — Figure S3. [file CNS-30-e14886-s004.pdf]

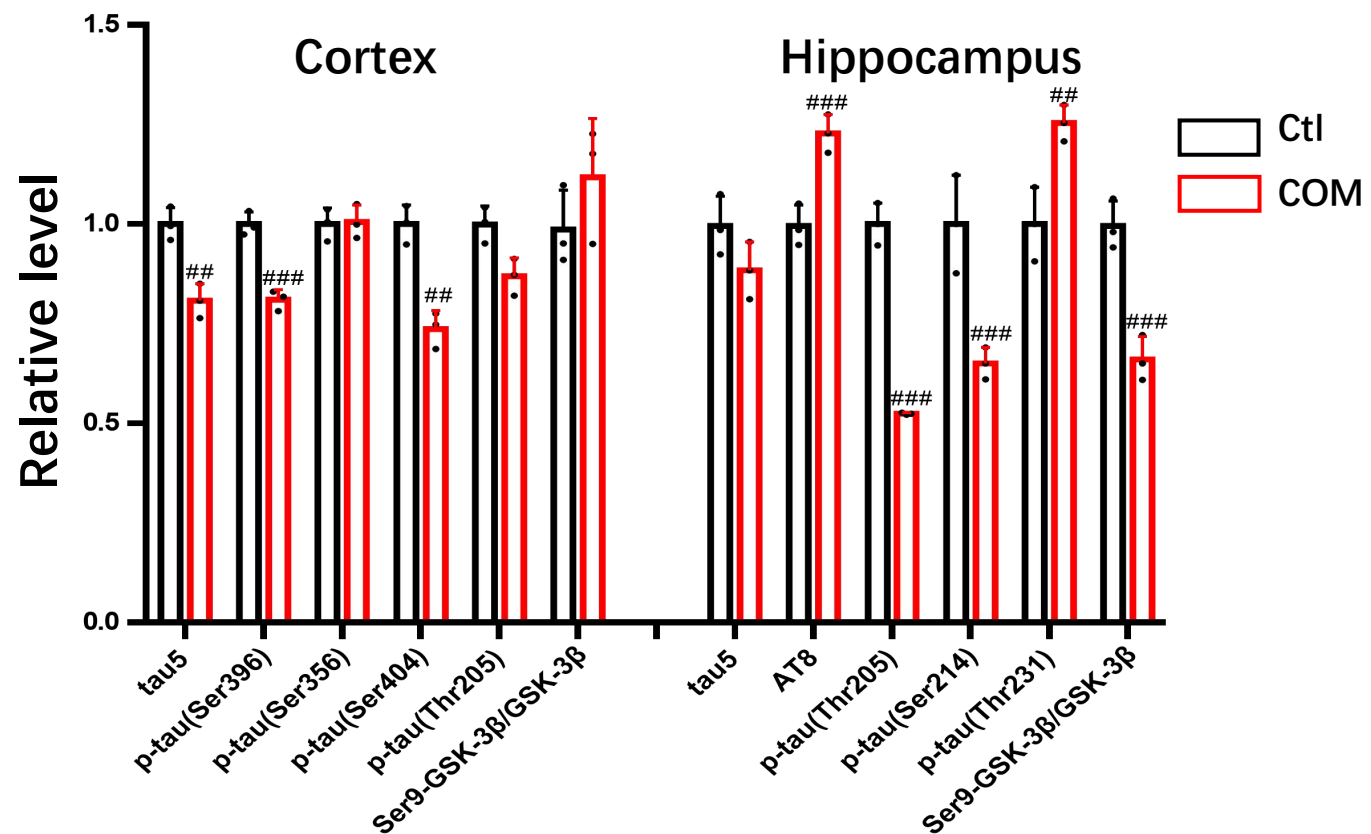

Supplement: Supplementary file 4 — Figure S4. [file CNS-30-e14886-s001.pdf]

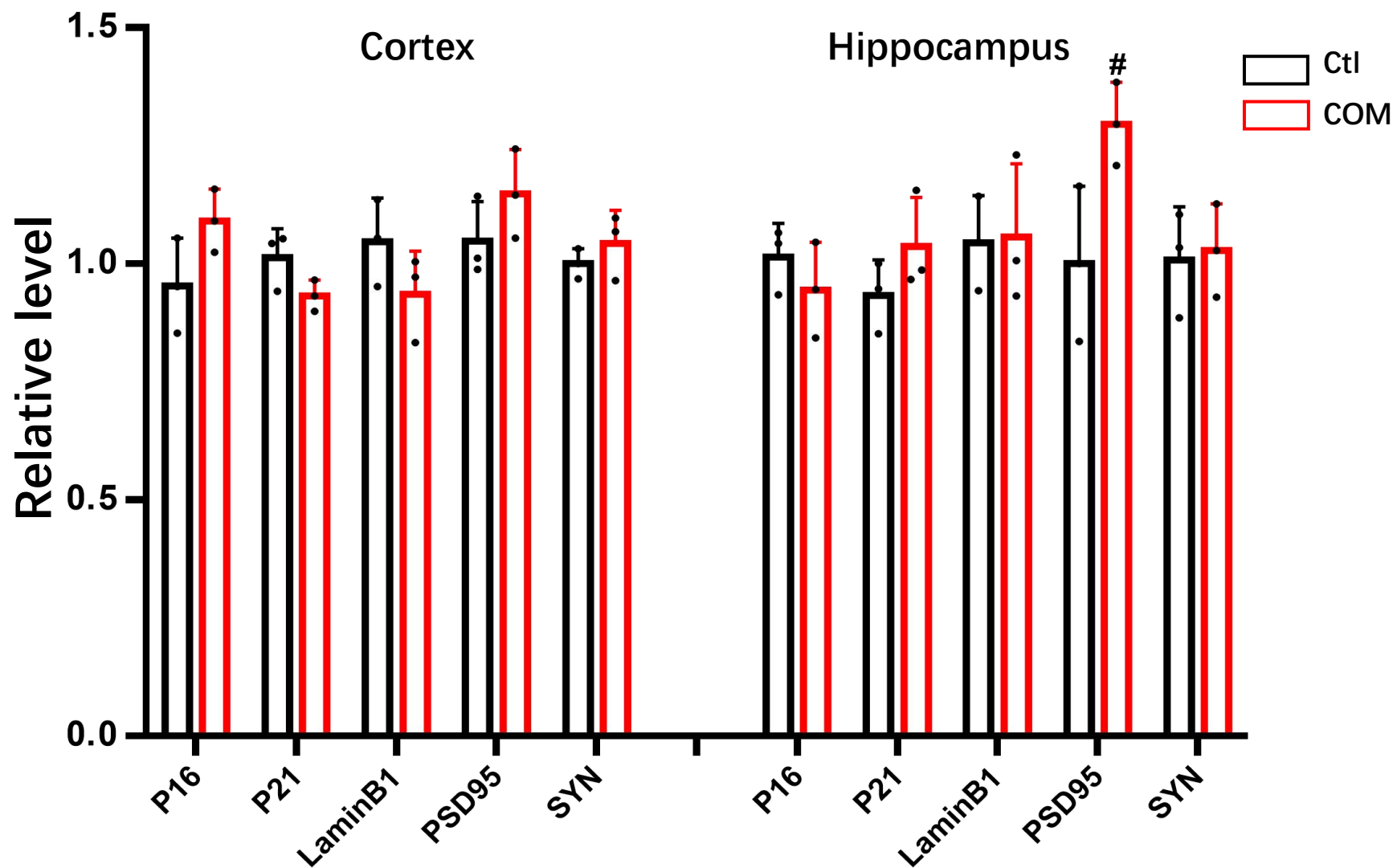

Supplement: Supplementary file 5 — Figure S5. [file CNS-30-e14886-s003.pdf]

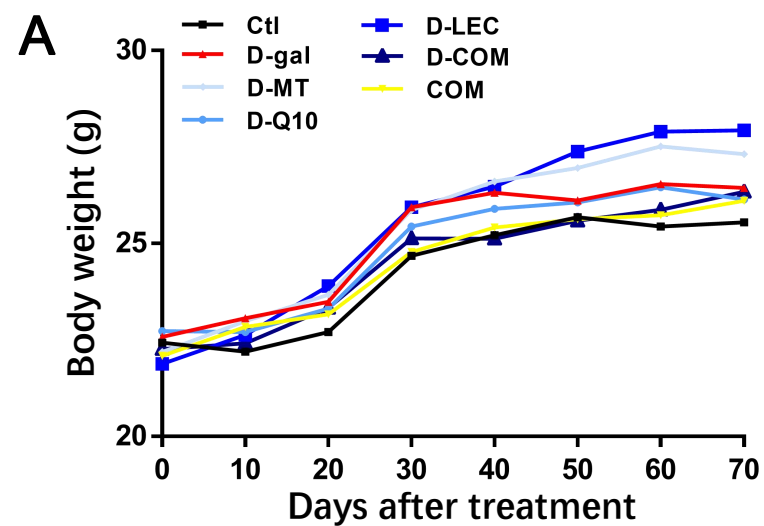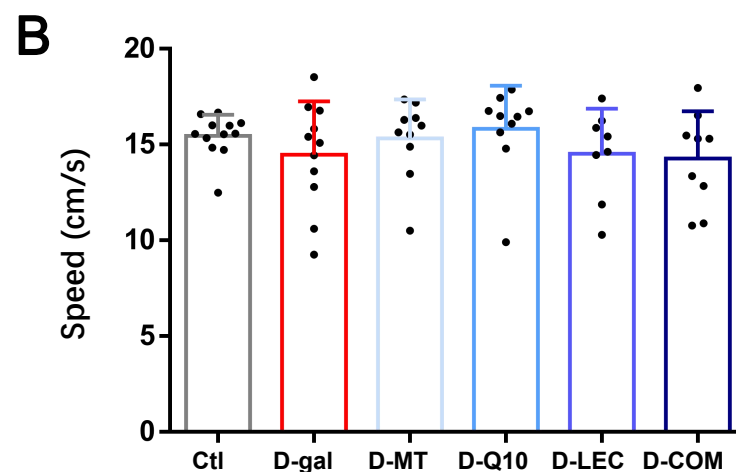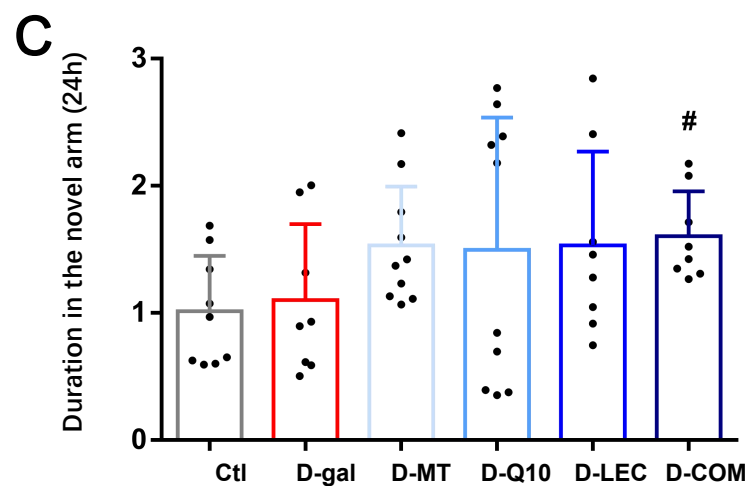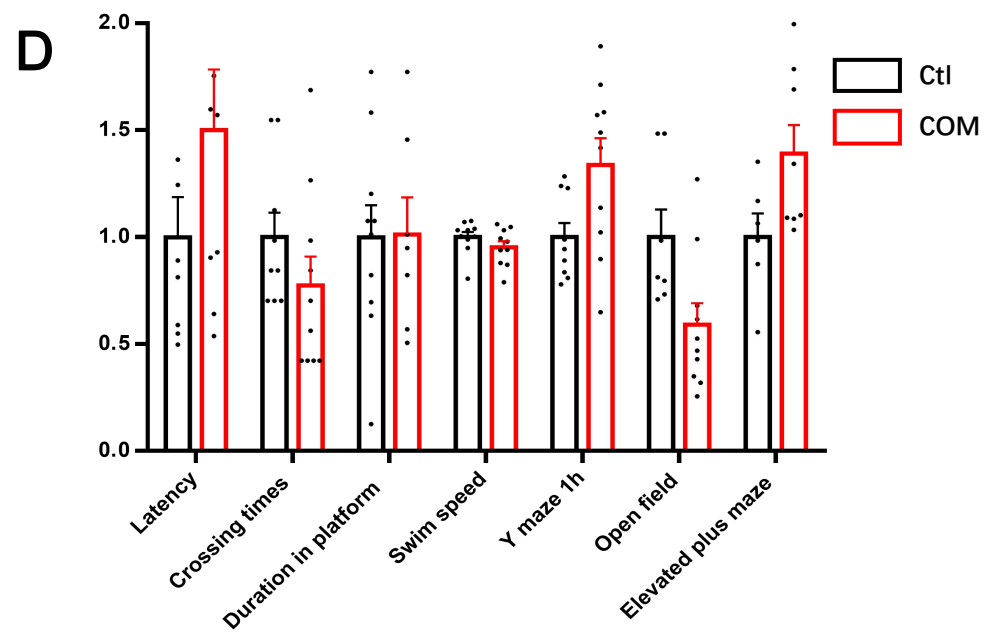

Supplement: Supplementary file 6 — Figure S6. [file CNS-30-e14886-s005.pdf]
